# Label-free photothermal disruption of cytotoxic aggregates rescues pathology in a *C. elegans* model of Huntington’s disease

**DOI:** 10.1038/s41598-021-98661-x

**Published:** 2021-10-05

**Authors:** Dmitry A. Nedosekin, TsungYen Chen, Srinivas Ayyadevara, Vladimir P. Zharov, Robert J. Shmookler Reis

**Affiliations:** 1 Arkansas Nanomedicine Center, University of Arkansas for Medical Sciences, Little Rock, AR 72205 USA; 2 College of Medicine, University of Arkansas for Medical Sciences, Little Rock, AR 72205 USA; 3 Carle Foundation Hospital, University of Illinois, Urbana, IL 61801 USA; 4 Department of Geriatrics, University of Arkansas for Medical Sciences, Little Rock, AR 72205 USA; 5 Central Arkansas Veterans Healthcare System, Little Rock, AR 72205 USA

**Keywords:** Biochemistry, Biotechnology, Molecular biology, Neuroscience, Medical research, Molecular medicine, Neurology, Nanoscience and technology

## Abstract

Aggregation of proteins is a prominent hallmark of virtually all neurodegenerative disorders including Alzheimer’s, Parkinson’s and Huntington’s diseases. Little progress has been made in their treatment to slow or prevent the formation of aggregates by post-translational modification and regulation of cellular responses to misfolded proteins. Here, we introduce a label-free, laser-based photothermal treatment of polyglutamine (polyQ) aggregates in a *C. elegans* nematode model of huntingtin-like polyQ aggregation. As a proof of principle, we demonstrated that nanosecond laser pulse-induced local photothermal heating can directly disrupt the aggregates so as to delay their accumulation, maintain motility, and extend the lifespan of treated nematodes. These beneficial effects were validated by confocal photothermal, fluorescence, and video imaging. The results obtained demonstrate that our theranostics platform, integrating photothermal therapy without drugs or other chemicals, combined with advanced imaging to monitor photothermal ablation of aggregates, initiates systemic recovery and thus validates the concept of aggregate-disruption treatments for neurodegenerative diseases in humans.

## Introduction

Abnormal protein aggregation is a key diagnostic feature and therapeutic target of age-progressive neuropathies including Huntington’s, Parkinson’s, and Alzheimer’s diseases^1–3^. The specific causes of protein damage and aggregate accumulation may vary, and can include genetic mutations, protein post-translational modifications (including oxidative damage, acetylation, and phosphorylation), and defective mechanisms of protein degradation, recycling, and refolding^4–8^. As these illnesses progress, protein aggregates are thought to overwhelm the normal cellular clearance mechanisms for misfolded proteins, which comprise chaperone-assisted refolding, the ubiquitin–proteasome system, and autophagy^9–11^. The resulting cytoplasmic, intra-nuclear, and pericellular inclusions then contribute to cell dysfunction and eventually death, via complex mechanisms^12,13^.

Huntington’s disease is a debilitating genetic illness which causes psychological instability as well as motor and cognitive disturbances^14^. Huntingtin (Htt) is a protein chiefly expressed in neurons and critical to normal development, although its role is not fully understood^6^. Some evidence suggests that formation of protein aggregates may be a protective mechanism against toxic mutant Htt occurring in a soluble form; however, accumulation of large, insoluble aggregates is ultimately cytotoxic as well^15,16^.

The genetically encoded trigger for Huntington's disease (HD) is an abnormally long glutamine-repeat tract (polyglutamine, or polyQ) exceeding 39 glutamine residues, located at the N terminus of Htt^17^. When abnormal Htt is being processed, expressed, and subsequently recycled and digested by the cell, the abnormally long N-terminal Htt fragments coalesce into a relatively insoluable *β*-sheet conformation that has a higher affinity for other unstructured hydrophobic polypeptides, including polyQ tracts. This results in precipitation and deposition of aggregates within cells^18^, which grow over time as the result of protein accumulation to the point that affected cells can no longer clear these particles by autophagy^15^. These events lead to cellular dysfunction and disruption of the tissues as a whole.

In humans, polyQ aggregates mainly deposit in neuronal tissues and this has been a focal point of research^15,19^. However, these aggregates also occur to a lesser extent within muscular, endocrine, and digestive tissues; evidence from humans and transgenic mouse models indicates that individuals with neurological HD traits also have decreased muscle development and mass, evident even in utero^20^.

Transgenic *C. elegans* models of protein aggregation provide valuable insights into age-dependent accumulation of damaged proteins, aggregation conditions, and mechanisms^3,7,10,21^. Additionally, influences of various genes and experimental therapies, including chemicals directly interacting with protein aggregates, can be studied in a genetically tractable organism under constant environmental conditions^22^. To date, no intervention has yet been shown to directly remove previously-deposited aggregates, or to disrupt them so as to allow their removal by cellular defense mechanisms, and thus restore normal functioning. The impact of aggregate disruption on subsequent cell clearance, and its impact on restoration of biological functions, have not previously been studied.

Although hormones, stress inhibitors, and autophagy inducers have been reported to prevent or delay aggregate formation and functional declines, these approaches all employed chemical agents or manipulations of the microenvironment that interfere with natural processes in cells^4,23–27^. Post-translational modifications and the use of chaperones affecting conformation of the mutated proteins have also been studied^15^.

The laser has revolutionized medical diagnosis and therapy^28^. We and others pioneered in 2003 photothermal (PT) therapy using pulse^29^ and continuous-wave^30^ lasers, with nanoparticles^31^ and intrinsic PT-contrast moieties (e.g., melanin)^32^, respectively. Subsequently, the potential of PT therapy was explored for selective treatment of cancer, infections, sickled erythrocytes, and cardiovascular disorders including clinically demonstrated 1000-fold elevation in the sensitivity and label-free killing of cancer cells in melanoma patients in vivo^32^. However, this is the first report of the successful application of PT therapy to the treatment of neurodegeneration-associated aggregates.

We here introduce a novel therapy, in vivo theranostics of protein aggregates, which integrates combined optical (PT and fluorescence) imaging of aggregates, PT disruption of pre-existing protein aggregates at the level of single cells or whole organisms, and imaging to monitor the efficacy of PT treatment. Label-free PT ablation also provides a unique opportunity to study basic cell biochemistry without the use of biologically active chemicals. In this study, we used *C. elegans* strain AM141, which expresses a 40-glutamine (Q40) tract fused in-frame to yellow fluorescent protein (YFP reporter), driven by a muscle-specific promoter (*unc-54p*) that normally drives myosin heavy chain synthesis in muscle cells. This transgenic nematode model forms fluorescent protein aggregates in four longitudinal stripes of body-wall muscle and becomes progressively paralyzed as it ages, culminating in death at ~ 14 days of age (well in advance of the normal wild-type lifespan of ~ 20 days at 20 °C). Visible-range lasers were utilized in this novel theranostics study, under conditions that can be replicated fairly easily—demonstrating the therapeutic potential of this technique.

## Results

### Principle of pulse PT therapy of protein aggregates

**Figure 1:**
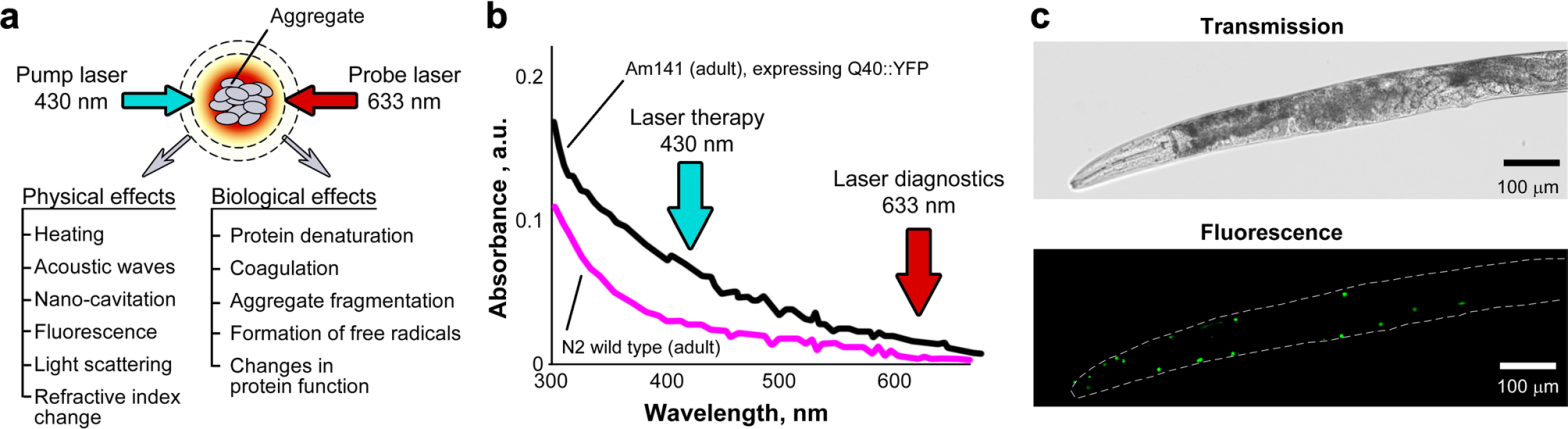
Laser-based theranostics of protein aggregates. (**a**) Principle of theranostics based on controllable PT and accompanying phenomena, both physical (e.g., acoustic and nanobubble) and biological (e.g., denaturation). (**b**) Laser excitation at two wavelengths for therapy (430 nm) and monitoring of resultant effects (633 nm). (**c**) Bright-field (transmission, top panel) and fluorescence microscopy (bottom) of a day-4 adult AM141 nematode showing Q40::YFP aggregates (green foci).

The irradiation of light-absorbing proteins with laser pulses in the visible spectral range leads to optical protein excitation followed by rapid non-radiative relaxation of absorbed energy as heat. Local protein heating is accompanied by physical and biological effects such as the generation of acoustic waves (photoacoustic effect), shock waves, and nanobubbles, leading to localized protein denaturation and ultimately disintegration through thermal and mechanical stresses (Fig. 1a). These effects alone or in combination may be accompanied by optical phenomena such as light scattering, fluorescence, secondary infrared radiation, and refractive-index variation, which can be used to monitor protein aggregates and to control the treatment parameters. In particular, laser pump pulse-induced refractive index changes are detected through defocusing of the second collinear continuous wave (CW) probe beam at 633 nm (Fig. 1b), leading to a reduction in probe-beam intensity detected by a photodiode fitted with a pinhole (thermolens effect)^33–36^. Using a pulse-focused laser beam, we limit laser-induced thermal effects to polyQ aggregates only (typically 4–8 µm in diameter) in order to minimize damage to surrounding healthy cells.

Full laser treatment of all visible aggregates in each worm (Fig. 1c) was performed on AM141 worms at post-hatch day 4 (group 1) or day 8 (group 2). For treatment, nematodes were anesthetized in fresh 0.3% sodium azide (NaN_3_) solution, and transferred to a glass coverslip mounted on the XY microscope stage. Each polyQ aggregate was irradiated with ~ 30 laser pulses at wavelength 430 nm and pulse energy of 1 µJ with the 100x objective, or 4 µJ with the 40x objective. All nematodes in the control group (group 3) were anesthetized and kept on a glass slide for the same duration but without laser treatment. Finally, we tested asymmetric/hemilateral therapy (group 4) with laser treatment delivered only to the aggregates on one side of each worm. Each group initially comprised 20 worms, of which 1–3 were censored (excluded for reasons unrelated to treatment).

### Localized laser disruption of polyQ aggregates

**Figure 2:**
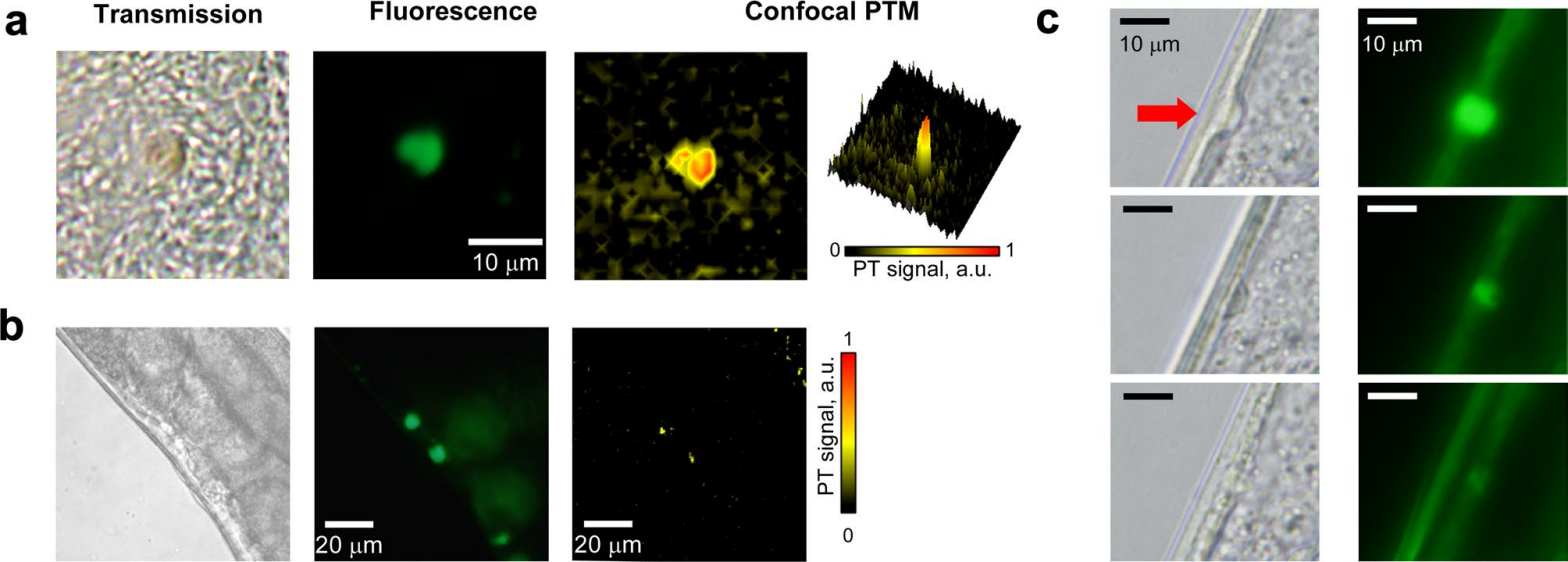
High resolution imaging of polyQ-YFP aggregates using transmission, fluorescence (YFP), and confocal PTM modalities. (**a**) Ex vivo, aggregates were separated from crushed worms prior to laser exposure. (**b**) In vivo, within a live worm. (**c**) Decreased fluorescence and changes in shape of a protein aggregate after laser therapy in vivo (top row—before therapy; middle and bottom rows—after 20 and 30 laser pulses, respectively, at 430 nm, 1 µJ/pulse; ×100 focusing objective).

In principle, laser exposure can lead to both highly localized thermal denaturation of proteins, and indirect physical damage due to pressure waves. To evaluate this hypothesis, we analyzed individual aggregates in live nematodes, or isolated from worm lysates, using confocal PT microscopy (PTM) which is very sensitive to light absorption contrast and its changes^34–36^. Indeed, PTM imaging confirmed that local absorption of these aggregates is 6–10 times higher than that of cell cytoplasm in both ex vivo and in vivo comparisons (Fig. 2a,b). The high-resolution optical images of aggregates inside a live worm before and after laser therapy suggest both thermal and acoustic damage mechanisms, as some of the aggregates disappear or exhibit dramatic changes in shape (Fig. 2c). Eventually, besides direct interaction with proteins, laser pulses in the range of 420–510 nm bleached the YFP fluorochrome fused to polyQ tracts, allowing real-time tracking of therapeutic efficiency as aggregates are lased (SI Fig. 3). Thus, the nanosecond laser pulses provide both imaging and especially aggregate visualization and also a mechanical-disruption modality, in which polyQ-YFP particles are damaged by expansion of nano- and micro-bubbles within the aggregates (SI Fig. 4).

### Full PT therapy: lifespan and motility changes

**Figure 3:**
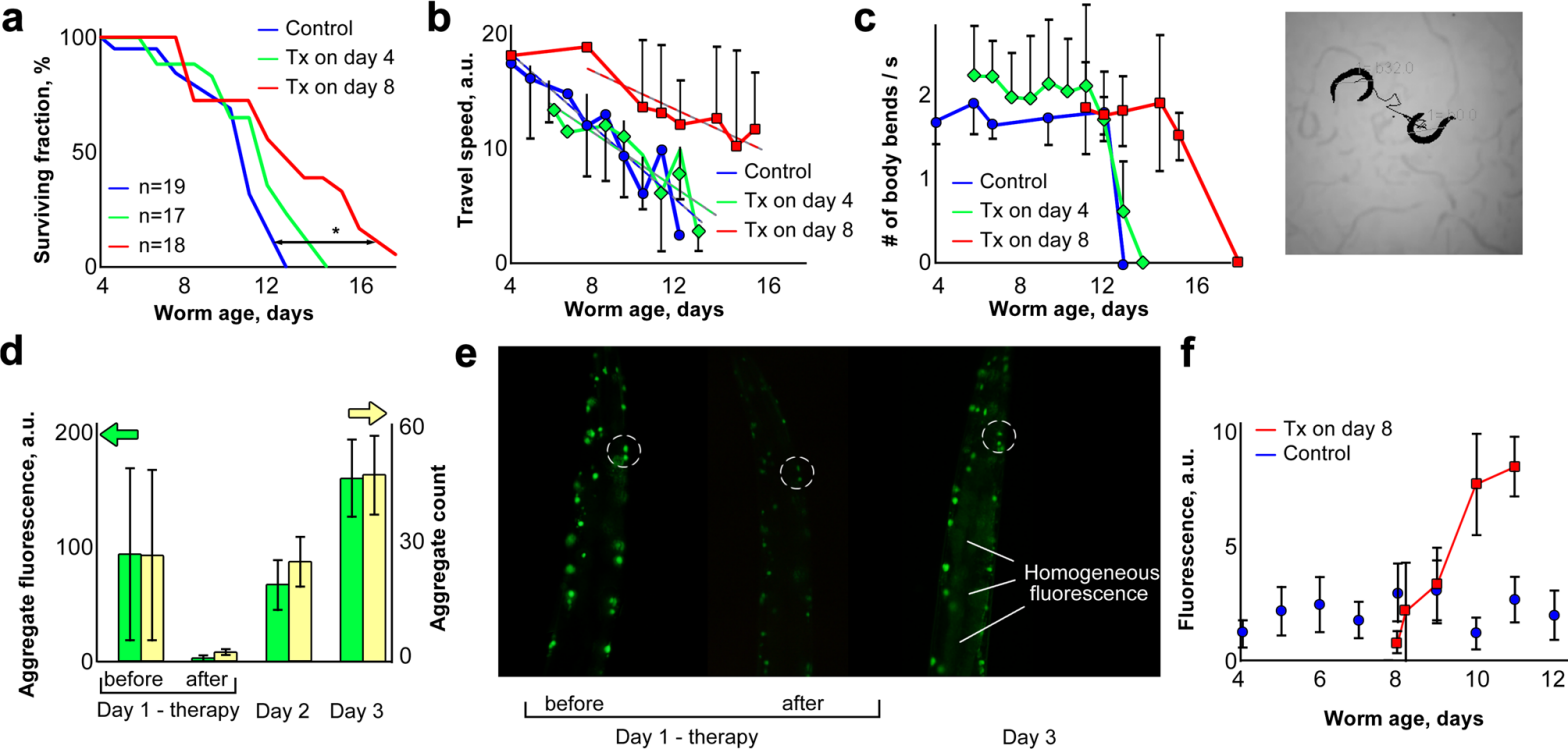
Laser disruption of polyQ::YFP aggregates, in 4 and 8 day-old AM141 nematodes. (**a**) Survivals for lifespan (*Cox–Mantel log-rank *p ≈ *0.003, day 8 laser vs. control). (**b**,**c**) Video analyses of worm-motility data (travel speed, flexion frequency). Each day-8 laser group differed from its control (**b**,  *p*< 6E–5; **c**, *p* < 0.01). Dashed lines in (**b**) show linear trend analysis for travel speed. Inset (right of **c**) shows a video frame with two worm positions connected by a midpoint trace. (**d**) Aggregate count (yellow) and total fluorescence (green) per worm. Each day-1/after differed from day-1/before (*t* test *p* < 0.05). Each day-2 and day-3 post-treatment group differed from day-1/after (*p* < 0.005). (**e**) Fluorescence images of laser-treated worms before, immediately after, and 48 h after laser therapy (dashed circles highlight two aggregates with post-therapy recovery of fluorescence). (**f**) Release of non-aggregate (diffuse) fluorescence. Error bars show SDs, unless omitted for clarity. Treatment differs from control at days 10–11 (combined *p* < 3E–5).

To assess the efficacy of PT therapy applied to adult worms at 4 and 8 days post-hatch, we monitored their subsequent survival and motility. Lifespan increased only slightly for worms treated on day 4 (*p* < 0.056), whereas laser treatment on day 8 was far more effective, extending mean survival by 20%, and 25th-percentile (longest-lived quartile) survival by 40% (Fig. 3a: *p* < 0.003 for log-rank comparison to controls). Similarly, motility benefited from PT therapy chiefly when worms were treated at 8 days; these worms remained active at least 21% (≥ 3 days) longer than the two other groups (Fig. 3b,c).

Laser bleaching of Q40::YFP molecules in aggregates was irreversible in vitro (at least over 5 days of monitoring). However, all treated worms recovered fluorescence of disrupted aggregates within 2–3 days, indicating that synthesis and re-aggregation of Q40::YFP protein continue unabated after therapy. The number and total fluorescence of aggregates reached pre-treatment levels within 2–3 days after laser therapy. New aggregates appeared, and some of those disrupted by treatment regained their former brightness (Fig. 3d,e), probably due to accrual of newly synthesized Q40::YFP protein. Direct comparison of laser-treated worms after recovery, with untreated controls, indicates that the overall fluorescence per aggregate did not exceed levels typical of AM141 adults (SI Fig. 5). However, a significant increase in diffuse background fluorescence was apparent in nematode tissues 2–3 days after laser therapy, in contrast to control worms for which background fluorescence did not change with age (Fig. 3e,f). In general, a diffuse background of unaggregated Q40::YFP protein is just barely above background in AM141 adults (after day 2.5 post-hatch), once aggregates have formed (Fig. 3e,f).

### Asymmetric PT therapy

**Figure 4:**
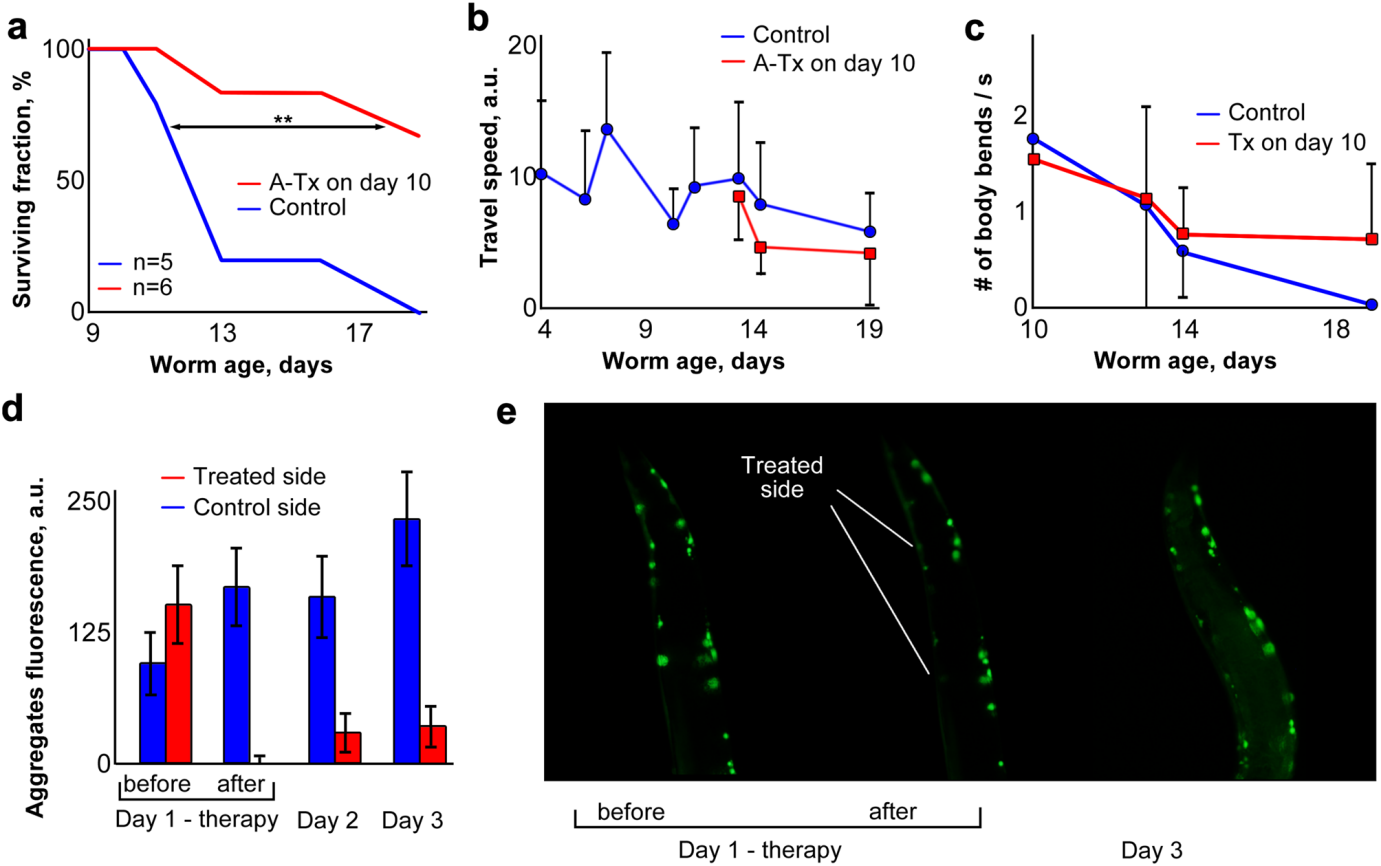
Asymmetric laser therapy in nematodes. (**a**) Lifespan survivals (**Cox–Mantel log-rank *p* < 0.013). (**b**,**c**) Video analysis of motility (travel speed; body-flexion frequency); differences were not significant. (**d**) Aggregate fluorescence intensity on treated vs. control sides. Treated side differed from control side at days 2 and 3, each *p* < 0.015 by paired *t* test; combined *p* < 1E–9. (**e**) Fluorescence imaging of laser-treated nematodes before, immediately after, and 48 h after laser treatment. Error bars in (**b**–**d**) indicate standard deviations; some are omitted for clarity in (**c**).

In 10-day-old worms that received laser treatment only on one side, lifespan increased significantly (*p* < 0.013) compared to control nematodes that received anesthesia only (Fig. 4a). As in the case of full-body therapy, velocity of movement and flexion frequency (Fig. 4b,c) were only modest prognostic indicators relative to survival (Fig. 4a), due in large part to day-to-day variation. In marked contrast to bilateral treatment (Fig. 3), the treated side did not return to pre-treatment aggregate fluorescence within 3 days (Fig. 4d) although aggregates on the control side showed the “normal” gradual increase in fluorescence observed in this strain. As for full-body therapy, hemilateral laser treatment was followed by a dramatic increase in diffuse background fluorescence (Fig. 4e).

The premise for performing asymmetric therapy was that treatment on only one side may produce asymmetric body-muscle function, i.e. the untreated side would serve as an internal control for functional impairment. Although these hemilaterally treated worms did not differ consistently or significantly from controls, in some instances we noted differences in body posture of the anesthetized worms: most non-paralyzed control worms under anesthesia were extended in a nearly-straight line whereas worms treated on only one side tended to flex under anesthesia with concavity on the treated side.

## Discussion

Many cell and whole-animal models of neuropathic aggregation have been developed, including *C. elegans* models of amyloidopathy elicited by expression of human “seed” proteins Aβ_1–42_, tau, TDP-43, α-synuclein, and polyglutamine tracts fused to yellow fluorescent protein^3,7,10,37–42^. We selected AM141, a disease-threshold model of polyglutamine tract expansion, because it displays age-progressive accrual of relatively large aggregates without transgene induction, resulting in functional motility impairment, reduced lifespan, and drug-induced diminution of aggregates^7,41,42^.

Using this model, we introduce a new therapeutic platform to demonstrate the feasibility of light-mediated disruption of aggregates. The platform employs advanced laser-based theranostics to target protein aggregates, integrating their optical visualization and image-guided PT treatment, with post-treatment imaging to monitor therapy efficacy. A novel laser pulse mode which we initially pioneered in 2003 for PT treatment of cancer^29,32^ and infections (e.g., *S. aureus*)^43^, was here applied to label-free treatment of protein aggregates typical of neurodegenerative diseases. Long CW exposures (5–10 min) were introduced by others^30^, but led to heat diffusion from the irradiated zone into the surrounding area, causing damage in this mode to a relatively large swath of healthy tissue (up to several mm from irradiation). We have similarly used tissue-destructive laser pulse energy to kill bacteria, *C. elegans* and mosquitos^34,43,44^, and super-resolution microscopy to detect and quantify malarial infection^45^, and endogenous chromophores in *C. elegans*, cultured cells, and circulating cancer cells in vivo^32,34,46^. Our PT pulse treatment was predicted to provide more localized target ablation without damage to surrounding healthy tissue due to negligible heat diffusion during the short laser pulses required (“thermal confinement conditions”^33^). Since the major component of polyQ aggregates is glutamine, with an absorption signature close to that of total protein (SI Fig. 2), there was limited benefit from spectral targeting, but we were able to reduce collateral damage by introducing tight beam focusing (~ 300 nm beam waist) and nanosecond-pulsed excitation.

For 5-ns laser pulses, the thermal diffusion distance was ~ 50 nm during the pulse ($l=\sqrt{4\alpha \tau }$, where α is thermal diffusivity [1.47 × 10^–7^ m^2^/s for water], and τ is pulse duration), which can be generally neglected. Finally, we anticipated that higher local density of the aggregates (compared to other cellular structures) might increase local optical density (i.e. light absorption), providing further target-specificity of the therapy.

The absorption of fluorescent markers is also somewhat unusual. At some point it attains saturation with all molecules excited, preventing further absorption. It will not work for PT therapy since all the molecules will be excited, will not absorb any more laser energy, and will not release any heat. We experimented briefly with YFP-specific laser wavelengths, but they were also absorbed by nearby cytochromes.

Specifically, we demonstrated that laser disruption of polyQ::YFP protein aggregates significantly delays the onset of paralysis and extends the lifespan of transgenic nematodes expressing a polyglutamine array in body-wall muscle. Laser-treated worms live substantially and significantly longer than untreated controls (*p* < 0.003), notwithstanding the expected harmful effects of such treatment. No deleterious effects of laser therapy were noted, and laser-treated worms at all ages were more mobile than same-age controls, and maintained that motility much longer than mock-treated worms. The similar efficacy of full-body and hemilateral therapies implies that aggregate disruption by laser pulses confers benefits that extend beyond the treated muscle cells. This is a remarkable and unforeseen observation that warrants further study.

Thus, the nanosecond-pulse laser treatment not only dramatically reduces imaging and visualization costs relative to other (e.g., femtosecond) laser sources, or use of a two-photon absorption mode, but also allows a mechanical-disruption modality, in which polyQ-YFP particles are disrupted by micro- and nanobubbles expanding within aggregates (SI Fig. 4).

It is puzzling that laser therapy was most beneficial later in life, i.e. laser ablation at 8 days of age was far more effective than at day 4, to preserve motility and reduce subsequent mortality. This may reflect the transient nature of the therapy—it removes aggregates but does not prevent their recurrance. Alternatively, large aggregates that accrue by day 8 may be more rigid and fragile, and thus more susceptible to laser ablation, than smaller, less mature aggregates. It is possible, but as yet untested, that repetition of laser treatments may provide additive benefit to treated tissues. Planned future studies will test methods to target and disrupt aggregates based on their unique biochemical properties rather than by manually targeting them with highly focused lasers. Additional studies will be needed to identify local and systemic mechanisms of action, including further comparisons with a variety of healthy nematodes. The normal decline with age, and high variance, of travel speed and body-flexion frequency may limit their prognostic value. Statistical testing by “trend analysis”, appropriate to these studies, has the greatest likelihood of showing significant change in either parameter for worms treated at day 8 or later.

The results presented here suggest clinical potential for photothermal disruption of protein aggregates in human neurodegenerative diseases, considering the relatively low laser energy required. Input energy requirements are further reduced if PT energy is directed at antibody-coated gold nanoparticles, some of which have been approved for clinical trials^46^, and which have light absorption 1–2 orders of magnitude greater than aggregates themselves, far exceeding protein or chemical targets. Alternatively, laser radiation can be delivered to deep pathological zones with minimal invasiveness using fiber optics as currently employed for cancer therapy. The current study serves as a proof-of-principle demonstration that debilitating protein-aggregation diseases may be alleviated or reversed by targeted disruption of aggregate foci.

## Methods

### Experimental animal strain

*C. elegans* strain AM141 was supplied by the Caenorhabditis Genetics Center (CGC, Minneapolis, MN) and maintained at 20 °C on 0.6% peptone NGM agar plates seeded with *E. coli* strain OP50^10^. AM141 worms express the *unc-54p*::Q40::YFP transgene in body-wall muscle cells, beginning immediately after hatching. Q40::YFP protein is initially soluble and diffuse in the cytoplasm, but is almost entirely aggregated into puncta by early adulthood (days 3–5 post-hatch).

### Laser platform for disruption of Q40::YFP aggregates

The laser therapy system was built on the foundation of an Olympus IX81 microscope equipped with a tunable laser-based optical parametric oscillator (OPO, Opolette HR 355 LD, OPOTEK, Carlsbad, CA) having a spectral range of 410–2200 nm, 5 ns pulse width, and 100 Hz pulse-repetition rate (see Supplementary Information, SI Fig. 1). In PT therapy mode, a laser beam (430 nm), was focused into a sample through a 100 × oil objective (DPlan100, Olympus Inc.) or an Olympus 40× objective. The energy of each OPO pulse was 1 μJ through the 100× objective, or 4 μJ after the 40× objective, controlled by an energy meter (PE10-SH, OPHIR, Logan, UT). For simultaneous YFP imaging and ablation therapy, a Hg-lamp light was filtered (FF01-480/17, Semrock Inc) and combined with laser light on a dichroic mirror (FF495-Di03, Semrock Inc) using a double lamp housing adapter (U-DULHA, Olympus). Fluorescence filter set FITC-3540B with excitation filter removed was installed in the microscope. A laser shutter (TSC001, Thorlabs, Newton, NJ) switched the laser beam ON for 20–40 pulses of 0.1 s each. The sample was manually scanned using a motorized translation stage (H117 ProScan II, Prior Scientific, Rockland, MA).

### Experimental design

AM141 worms were synchronized by alkaline-detergent lysis of gravid hermaphrodites, and transfer of their unlaid eggs onto 60-mm culture plates seeded with *E. coli* OP50 bacteria. Full laser therapy (targeting all visible aggregates in each worm [Fig. 1]) was performed on AM141 worms at day 4 post-hatch (group 1) or day 8 (group 2). Nematodes were anesthetized in fresh 0.3% sodium azide (NaN_3_) solution, and transferred to glass coverslips mounted on the XY microscope stage. To reduce anesthetic duration, only one nematode was treated at a time and quickly rinsed in phosphate-buffered saline at the end of the procedure. Each polyQ aggregate was irradiated with 20–40 laser pulses (1 or 4 µJ/pulse, depending on the objective; see preceding section), where the exact number of pulses depended on aggregate size, shape, and residual of YFP fluorescence after laser ablation. All nematodes in the control group (group 3) were anesthetized and kept on a glass slide as described above for the same duration but without laser treatment. Finally, we tested asymmetric/hemilateral therapy (group 4) with laser treatment delivered only to the aggregates on one side of each worm. After the laser therapy, all nematodes were transferred onto individual agar plates seeded with bacteria. The initial size of each group was 20 worms (individuals successfully transferred to agar plates after laser treatment). However, nematodes lost, stranded, or accidentally damaged in the days following treatment were censored from the experiment, reducing the final count to 17–19 per group.

### Lifespan survival assays

Laser-treated and control nematodes were scored daily for spontaneous movement or touch response. Completely unresponsive worms were counted as dead, unless censored (deaths due to misadventure or internal hatching). Significance of survival differences was assessed by Cox-Mantel log-rank tests.

### Analysis of nematode motility

Motility of the worms was analyzed using video recordings of worm movements in a drop of water on the surface of an agar plate. A Focus camera mounted on an Olympus microscope was connected to an iPad used for video capture. Video files were decompressed by ImageJ software (NIH) and analyzed using the *wrmTrack* plugin (see http://www.phage.dk/plugins/wrmtrck.html for details). Both travel speed and the frequency of body flexion were calculated for each moving nematode and averaged for the population. Dead or paralyzed nematodes were censored from motility calculations on the day movements ceased, and movement was characterized for the remaining worms only.

### Quantitation of Q40::YFP aggregates

Fluorescence microscopy (FITC-3540B filter set, Semrock Inc, Rochester, NY) was used to count Q40::YFP aggregates and to calculate an integrated intensity score. Images were acquired by an Olympus IX81 fluorescence microscope equipped with DP72 camera (Olympus Inc., Center Valley, PA). Automated image analysis was performed with custom macros written for ImageJ (NIH) to adjust intensity thresholding and for particle-counting.

### UV–Vis spectroscopy of purified protein aggregates

UV–Vis spectra of purified protein aggregates were obtained with a NanoDrop 8000 spectrophotometer (Thermo Fisher Scientific Inc., Wilmington, DE; see Supplemental Information for sample preparation). After calibration, 1-µl samples were loaded and spectra collected across wavelengths 200–750 nm.

### In vivo confocal photothermal microscopy of protein aggregates

The laser platform developed for laser ablation of proteins was converted into a confocal photothermal (PT) microscope as described elsewhere^34^ by combining the excitation laser beam with a collinear low-power probe laser (633-nm, He–Ne laser, model 117A, Spectra-Physics, Santa Clara, CA). Light absorption by aggregates resulted in formation of a local refractive-index change due to increase in local temperature. Probe beam modulation by this PT phenomenon was monitored with a photodetector (PDA36A, 40 dB amplification, ThorLabs, Newton, NJ) shielded with a narrow-bandpass filter (central wavelength 633 nm, spectral bandwidth 12.5 nm, LL01-633-12.5, Semrock, Inc., Rochester, NY). Data acquisition and system synchronization were performed using a high-speed (200 MHz) analog-to-digital converter board PCI-5124, 12-bit card, 128 MB of memory (National Instruments, Inc., Austin, TX).

### Statistical analyses

All results are expressed as means ± standard deviation (SD), indicated by error bars. Results shown are supported by at least 3 independent experiments to ensure reproducibility. Statistica 5.11 (StatSoft, Inc.), MATLAB 7.0.1 (MathWorks), and LabVIEW (National Instruments) were used for statistical tests. All *p* values shown reflect 2-tailed test results, although 1-tailed tests are sufficient when direction of change is known. Significance of survival differences was assessed by Cox-Mantel log-rank tests of full plots.

## Supplementary Information

Supplementary Information.
