# Supplementary material for: Label-free photothermal disruption of cytotoxic aggregates rescues pathology in a C. elegans model of Huntington’s disease
Source: Sci Rep. 2021 Oct 5;11:19732. doi: 10.1038/s41598-021-98661-x (PMC8492664; doi:10.1038/s41598-021-98661-x)
Supplement: Supplementary file 1 — Supplementary Information. [file 41598_2021_98661_MOESM1_ESM.pdf]

SUPPLEMENTARY INFORMATION

Label-free photothermal disruption of cytotoxic aggregates rescues pathology in a *C. elegans* model of Huntington's disease

D.A. Nedosekin, T.-Y. Chen, S. Ayyadevara, V.P. Zharov, R.J. Shmookler Reis

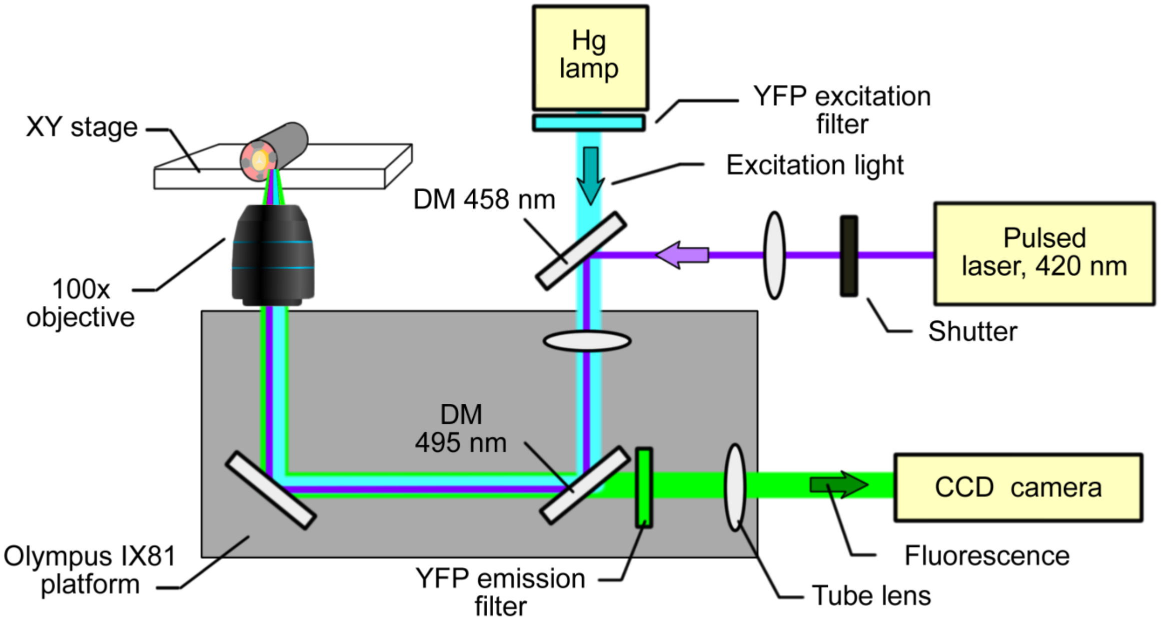

SI Figure 1. Schematics of a fluorescence microscope with integrated nanosecond-pulsed laser source for point disruption of aggregates with real-time fluorescence microscopy imaging for therapy guidance.

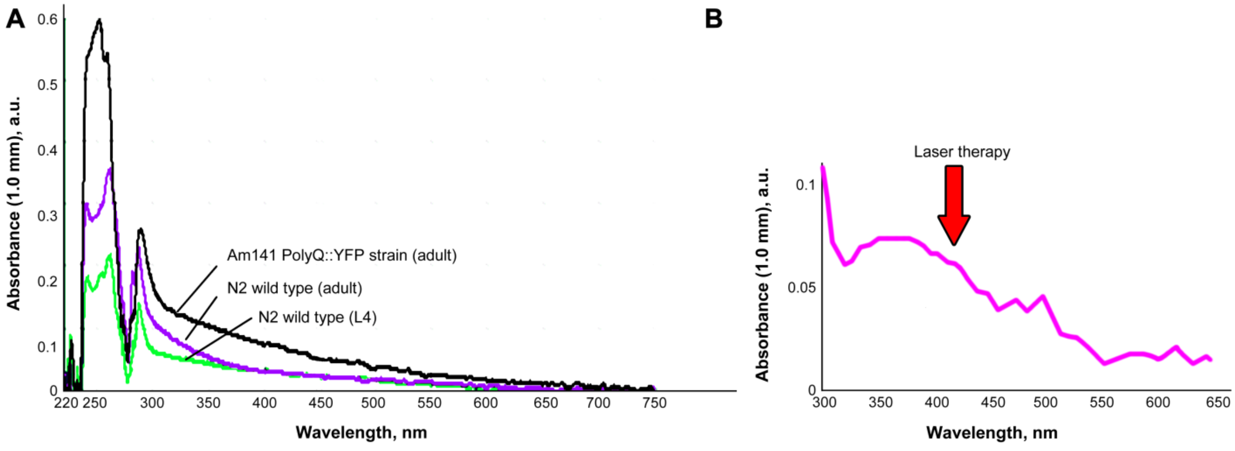

SI Figure 2. **A**, UV-Vis absorption spectra of dense aggregates isolated from the AM141 stain of *C. elegans* (adults, black line). Spectra of N2 wild-type adults (purple line) and immature/L4 N2 worms (green line) are shown as controls. Aggregates were prepared in phosphate-buffered saline and analyzed in a NanoDrop spectrophotometer (1.0-mm optical path). **B,** Spectral difference between AM141 and N2 adult worms indicates polyQ-YFP absorbance. Red arrow indicates the laser wavelength selected for therapy.

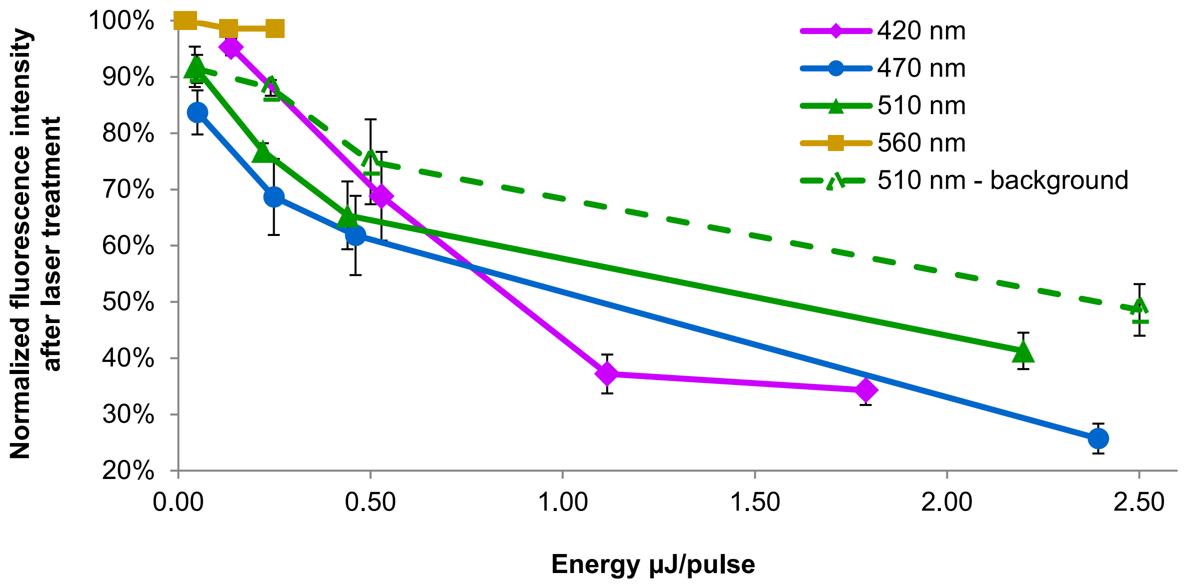

SI Figure 3. Changes in intensity of aggregate fluorescence after therapy using 10-nanosecond laser pulses.

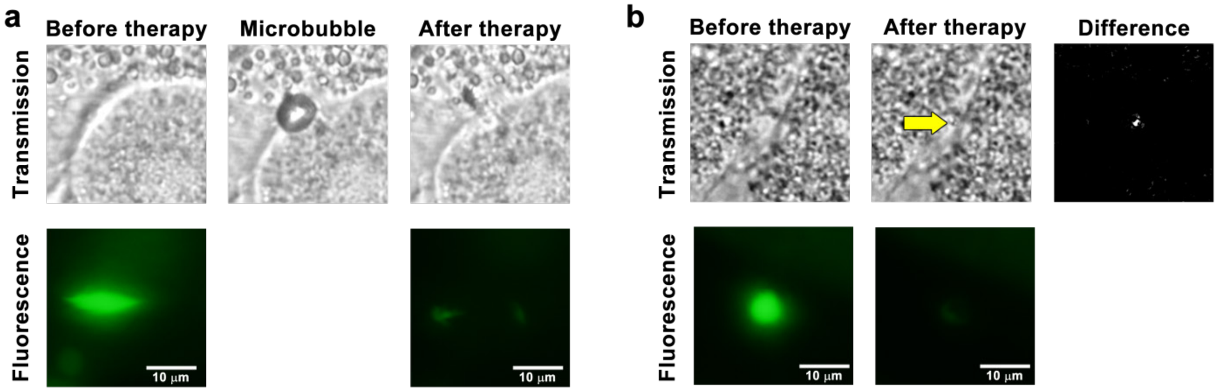

SI Figure 4. Mechanical disruption of polyQ-YFP aggregates by micro- and nano-sized air bubbles. **a**, Transient microbubble formed at high laser energy. The image after therapy indicates possible tissue damage. **b**, Transient sub-micrometer air bubble. Right panel (labeled *Difference*) presents an image calculated as the pixel-specific difference between the left and central images. The yellow arrow indicates a tiny air bubble formed within the sample. Laser parameters: 420 nm, 10 (**a**) and 1 (**b**) µJ per pulse; 100× focusing objective).

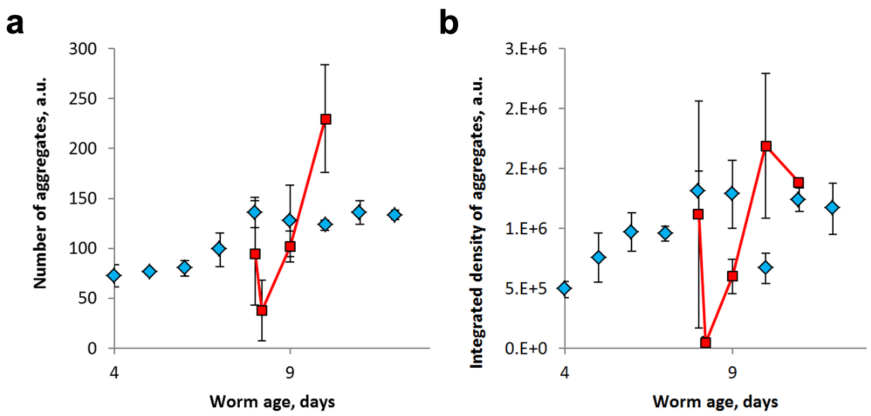

SI Figure 5. Comparison of fluorescence of polyQ-YFP aggregates in control (blue dots) and laser-treated worms (red dots): **a**, Total number of aggregates; **b**, Integrated intensity of all aggregates per worm.

UV-Vis spectroscopy of isolated and purified worm aggregates: sample preparation

AM141 worms were synchronized and flash-frozen in liquid nitrogen at the time of harvest. They were then pulverized in a dry ice-cooled mortar and suspended in lysis buffer (20-mM HEPES at pH 7.4, 0.3-M NaCl, 2-mM $MgCl_2$, 1% NP-40 (w/v), and phosphatase/protease inhibitors [CalBiochem]). Suspensions are then sonicated for 10 seconds followed by 10 seconds on ice. After centrifugation (5 min., 2000 x g) to remove debris, organelles, and particulates, the protein concentration was determined (Bradford Protein Assay, Bio-Rad). Protein samples were centrifuged at 14,000 x g for 15 minutes and supernatants were removed. Pellets were then suspended in 0.1-M HEPES buffer containing 5-mM EDTA and protease inhibitors.

Analysis of worm motility using ImageJ with wrmTrack plugin

To optimize video recording of worm movements, we selected clear patches on agar plates (to minimize artifacts). Several video recordings, each of ~30 s duration, were acquired for each worm using the same magnification. Scale calibration was performed by acquiring an image of a ruler in each experiment.

Acquired video files were converted to archos 504/604 Movie (*avi) format at 25 frames/sec (Any video converter, http://download.cnet.com/Any-Video-Converter-Freeware/3000-2194_4-10661456.html). For ImageJ analysis, files were decompressed using Virtual Dub software (http://www.virtualdub.org/). Video artifacts were manually removed by cropping out selected frames.

wrmTrack analysis has been described in detail elsewhere. In brief, standard ImageJ procedures are used to prepare the video file for analysis. First, the decompressed AVI file is converted to grayscale. Max intensity Z-stack is then created. Using frame 31 as a reference, the difference images are calculated for each frame giving a dark background and the worm in white.

We used the following settings to run wrmTrack analysis:

minSize	200	pixels^2
maxSize	90000	pixels^2
maxVelocity	15	pixels/frame
maxAreaChange	30	%
MinTrackLength	200	frames
bendThreshold	1	
binSize	0	0 = disable
RawData	0	0 = OFF
bendDetect	1	1 = Angle
FPS	0	0 = load from file
ThreshMode	MaxEntropy	
